# Elucidating the multifaceted role of MGAT1 in hepatocellular carcinoma: integrative single-cell and spatial transcriptomics reveal novel therapeutic insights

**DOI:** 10.3389/fimmu.2024.1442722

**Published:** 2024-07-16

**Authors:** Yang Li, Yuan Chen, Danqiong Wang, Ling Wu, Tao Li, Na An, Haikun Yang

**Affiliations:** ^1^ Department of General Medicine, Shanxi Bethune Hospital, Shanxi Academy of Medical Sciences, Third Hospital of Shanxi Medical University, Tongji Shanxi Hospital, Taiyuan, China; ^2^ Department of Geriatrics, Tongji Hospital, Tongji Medical College, Huazhong University of Science and Technology, Wuhan, China; ^3^ Tumor Center, Shanxi Bethune Hospital, Shanxi Academy of Medical Sciences, Third Hospital of Shanxi Medical University, Tongji Shanxi Hospital, Taiyuan, China; ^4^ The Gastroenterology Department, Shanxi Provincial People Hospital, Taiyuan, China

**Keywords:** spatial transcriptomics, single-cell RNA sequencing, hepatocellular carcinoma, pathogenesis, multi-omics data, tumor heterogeneity, tumor microenvironment, novel biomarkers

## Abstract

**Background:**

Glycosyltransferase-associated genes play a crucial role in hepatocellular carcinoma (HCC) pathogenesis. This study investigates their impact on the tumor microenvironment and molecular mechanisms, offering insights into innovative immunotherapeutic strategies for HCC.

**Methods:**

We utilized cutting-edge single-cell and spatial transcriptomics to examine HCC heterogeneity. Four single-cell scoring techniques were employed to evaluate glycosyltransferase genes. Spatial transcriptomic findings were validated, and bulk RNA-seq analysis was conducted to identify prognostic glycosyltransferase-related genes and potential immunotherapeutic targets. MGAT1’s role was further explored through various functional assays.

**Results:**

Our analysis revealed diverse cell subpopulations in HCC with distinct glycosyltransferase gene activities, particularly in macrophages. Key glycosyltransferase genes specific to macrophages were identified. Temporal analysis illustrated macrophage evolution during tumor progression, while spatial transcriptomics highlighted reduced expression of these genes in core tumor macrophages. Integrating scRNA-seq, bulk RNA-seq, and spatial transcriptomics, MGAT1 emerged as a promising therapeutic target, showing significant potential in HCC immunotherapy.

**Conclusion:**

This comprehensive study delves into glycosyltransferase-associated genes in HCC, elucidating their critical roles in cellular dynamics and immune cell interactions. Our findings open new avenues for immunotherapeutic interventions and personalized HCC management, pushing the boundaries of HCC immunotherapy.

## 1 Introduction

Hepatocellular carcinoma (HCC) is recognized as a predominant and formidable type of liver cancer (1, 2). It constitutes approximately 80% of all primary liver cancer cases worldwide, which equates to a staggering increase of over 5 million new patients each year (3–5). Notably, HCC is ranked fifth in terms of global incidence among all cancers yet occupies a troubling second position in cancer-related mortality, highlighting its bleak survival rates (6–9). This malignancy stands out among the top five most common cancers due to its concurrent rise in both occurrence and death rates (10). Exploration into the complex causes of HCC unveils a diverse array of factors including viral infections, genetic predispositions, environmental influences, and epigenetic modifications. A series of key biomarkers such as alpha-fetoprotein (AFP), GP73, FARSB, and serum miR-483-5p have been identified through the years, signifying significant advances in the detection, prognosis, and management of this tumor (11–15). Despite the availability of an extensive range of treatment options like surgery, radiation therapy, chemotherapy, and targeted interventions, the inherently aggressive and variable nature of HCC makes managing and predicting its course particularly challenging (16–18). This complexity persists despite advances in therapeutic strategies, underscoring the formidable challenges in treating HCC effectively (19).

Glycosyltransferases, comprising a broad spectrum of enzymes, facilitate glycosylation processes that are critical to the pathophysiological outcomes of various diseases (20). Glycosylation, an intricate post-translational mechanism affecting proteins and lipids, controls a range of cellular functions from cancerous growth and invasion to angiogenesis and the development of severe disorders (21, 22). Within the realm of cancer, abnormal glycosylation is recognized as a distinctive disruption, significantly affecting metastatic capabilities and the ability of cells to evade immune detection (23). Recent research highlights the complex relationships between aberrant glycosylation of proteins and alterations in malignant cellular behaviors (24). Furthermore, glycosyltransferases are pivotal in modulating how the immune system recognizes cellular exteriors and, together with cytokines from the immune microenvironment, regulate immune responses by influencing glycosyltransferase activities within cells and shaping the glycosylation patterns of IgG (25, 26). As the therapeutic significance of glycosyltransferases gains attention, exploring their regulatory complexities and features is becoming a cutting-edge area of focus, promising to revolutionize and enhance oncological treatment strategies (27, 28).

Macrophages, pivotal components of the immune system, excel in conducting phagocytosis of pathogens, compromised cells, and cancerous formations, thus maintaining immune balance and eliminating abnormal cellular elements (29, 30). In the cancerous environment, these guardians exhibit anti-tumor capabilities by efficiently recognizing and engulfing cancer cells (31). These macrophages are categorized into M1 and M2 phenotypes, with M1 macrophages actively resisting tumor growth and M2 macrophages, paradoxically, promoting it (32, 33). Within the chaotic realm of the tumor microenvironment, M2 macrophages play a significant role, secreting cytokines such as interleukin-10 (IL-10) and transforming growth factor-β (TGF-β). These substances suppress immune responses, weaken host defenses against cancer (34), and facilitate tumor evasion from immune surveillance (35). The prevalence and activity of macrophages carry significant prognostic implications for cancer outcomes (36). Therefore, detailed examination of macrophage roles in hepatocellular carcinoma is crucial, potentially revealing new paths to enhance and refine immunotherapeutic strategies.

Within the complex landscape of hepatocellular carcinoma (HCC), immune guardians continuously identify and engage aberrant cells, including cancerous ones (37). An intriguing tactic employed by certain cancers, particularly those of the liver, involves hijacking the PD-1/PD-L1 pathway to surreptitiously evade immune surveillance (38, 39). Central to this advancement are the anti-PD-1/PD-L1 agents, which adeptly modulate immune defenses against HCC. These agents disrupt the interaction between PD-1 and PD-L1, thereby reinforcing the immune defense against liver cancer (40). Specifically, these therapeutic agents enhance immune function by lifting the inhibitory effects on T-cells imposed by the PD-1 or PD-L1 proteins (41). Specifically, these therapeutic agents enhance immune function by lifting the inhibitory effects on T-cells imposed by the PD-1 or PD-L1 proteins (42–44). Furthermore, this category of drugs has demonstrated a remarkable capacity to boost overall immune strength, halting the aggressive spread of cancer cells and thus acting as guardians against disease progression (45). However, the variability in therapeutic outcomes highlights the diverse responses among patients. Consequently, the critical challenge now lies in identifying which HCC patients are likely to benefit most from immunotherapy, a key step towards improving prognostic accuracy.

While recent technological advancements have significantly enhanced our exploration of glycosyltransferases using sophisticated molecular biology techniques such as transcriptome and single-cell sequencing, the complex mechanisms through which they influence tumorigenesis remain largely mysterious. The complex cellular and immune environments within tumor landscapes (46)highlight the intricate interactions between macrophages and glycosyltransferases in cancer development. In our research, we sought to unravel the genomic patterns associated with macrophage- and glycosyltransferase-related genes in hepatocellular carcinoma (HCC). Despite the capabilities of advanced tools like single-cell sequencing, obtaining a spatially integrated understanding of the disease has been challenging. Our research thus combined single-cell with spatial transcriptome sequencing to deeply understand the interplay of glycosyltransferase-related genes within the immune context of HCC. Furthermore, we integrated insights from bulk transcriptomics to develop a robust prognostic framework, thereby enhancing the precision of therapeutic strategies in clinical settings for HCC. Through comprehensive sequencing, we have identified MGAT1 as a pivotal target for immunotherapy in HCC. This discovery is substantiated by a robust combination of bioinformatics analysis, cellular assays, and experimental validation. Our findings elucidate the regulatory roles of glycosyltransferases in HCC, offering a foundation for innovative immunotherapeutic and molecular strategies aimed at these enzymes in HCC management.

## 2 Materials and methods

### 2.1 Source of raw data

For our HCC single-cell sequencing investigation, we meticulously selected data from seven primary, untreated HCC samples located in GSE112271 of the GEO database. Concurrently, we obtained three corresponding normal tissue samples from GSE182159, each representing a different case. Insights into the anti-PD-1/PD-L1 immune response were garnered from the cohort detailed by Cho et al. in GSE126044. The spatial transcriptomic foundations, achieved via stRNA-seq for HCC on the 10x Visium platform, were sourced from GSE224411. A pivotal study titled “An integrative pan-cancer analysis of the molecular and biological features of glycosyltransferases” provided a collection of 185 glycosyltransferase-related genes crucial to our research (47). Moreover, we integrated bulk RNA-seq data of HCC from the TCGA cohort, consisting of 424 samples, available through the Xena portal (https://xena.ucsc.edu/). Additionally, disease-free survival data associated with TCGA were extracted from the cBioPortal repository (https://www.cbioportal.org).

### 2.2 Single-cell sequencing data processing

In our single-cell RNA sequencing (scRNA-seq) evaluation, we employed the R package “Seurat” for a meticulous cell-level analysis (48, 49). We filtered out cells exhibiting over 5% mitochondrial gene content or expressing between 200 and 2,500 genes to maintain data integrity. To address batch discrepancies between tumor and normal samples, we used the “harmony” R package. Following normalization with Seurat’s “NormalizeData” function, the data was transformed into Seurat objects. We identified the top 2000 variable genes using “FindVariableFeatures,” and then reduced dimensionality through Principal Component Analysis (PCA) using “RunPCA”. Significant principal components (PCs) were determined via JackStraw analysis, with selection based on variance ratios for subsequent cell clustering. Utilizing “FindNeighbors” and “FindClusters”, we clustered the data and visualized cell populations through the uniform manifold approximation and projection (UMAP) technique (50). For delineating cluster-specific genes, we applied a Wilcoxon rank-sum test with “FindAllMarkers” and “FindMarkers” from the “scran” R package (51), and annotated cell types using the CellMarker database (http://xteam.xbio.top/CellMarker/index.jsp).

Intercellular communication dynamics, particularly regarding HCC’s immune profile, were elucidated using the “CellChat” R package, which simulated communication probabilities based on gene expression and known ligand, receptor, and cofactor interactions (52). We analyzed the repertoire of 185 glycosyltransferase-associated genes across five scoring algorithms (AddModuleScore, AUCell, UCell, singscore), employing “SingleR’s” “AddModuleScore” for genome scoring based on mean gene expressions. The methodologies of “AUCell”, “UCell”, and “singscore” focused on gene enrichment ranking, unsupervised cell-type annotation, and functional activity quantification within single cells or samples. This multiplexed scoring approach enriched our analysis with robustness and depth.

For macrophage-related analyses, we used the “limma” R package to perform differential gene analysis, overlaying the results with the glycosyltransferase gene set to identify key macrophage-associated glycosyltransferase genes (53, 54). Pseudotime trajectory mapping, elucidating cellular evolution patterns pertinent to tumorigenesis, was achieved using the “Monocle” package. Additionally, “CellCall” revealed integrated intercellular communication networks, combining ligand-receptor dialogue and intracellular transcription factor dynamics to construct the L-R-TF axis, while integrating pathway activity assessments to identify cellular pathway alterations driven by intercellular communication.

### 2.3 Spatial transcriptome sequencing data processing

We processed and analyzed spatial transcriptomic data using the “Seurat” R package. The normalization and scaling of UMI counts were performed, with key features identified through the “SCTransform” procedure. Dimensionality reduction was conducted using the “RunPCA” method. Distinct subgroups and their respective genes were visualized using the “SpatialFeaturePlot” function. To explore cellular metabolic characteristics, we utilized the “scMetabolism” R package, employing the VISION algorithm to score each cell and determine metabolic pathway activity. This detailed metabolic analysis revealed the complex functionalities and variations within cells, providing a spatial and functional understanding of the tumor.

The “Monocle” R package enabled the temporal analysis of spatial transcriptomic data, uncovering the developmental and differentiation trajectories of spatially distinct cell clusters. Additionally, we employed the Python-based “Scanpy” suite, which integrates preprocessing, visualization, clustering, time-series extrapolation, and differential expression assessment, thereby enhancing the depth of single-cell analysis.

To add another layer of analysis, we used the “stlearn” tool from the University of Queensland’s Institute of Molecular Biosciences. This tool combines gene expression data, tissue morphology, and spatial coordinates, allowing for robust cell type identification, tissue-centric cellular reconstructions, evolutionary pathway inference, and identification of regions with significant intercellular interactions. The integration through “stlearn” provided profound insights into cellular interactions, enriched by ligand pair data, gene expression profiles, spatial topology, and nuances in spatial cellular distribution.

### 2.4 Spatial transcriptome data combined with single-cell sequencing data for deconvolution analysis

Deconvolution analysis, a technique for discerning cellular proportions within heterogeneous samples, attains enhanced precision by integrating single-cell and spatial transcriptomic datasets. Single-cell sequencing elucidates the cellular diversity within tissues, while spatial transcriptomics pinpoints the precise locations of these cells, capturing spatial complexities. By leveraging advanced deconvolution methods such as robust cell type decomposition (RCTD) available in the “spacerxr” R package, we gain unparalleled insights into the spatial intricacies and heterogeneity of tumors. Implementation begins with establishing a well-annotated single-cell transcriptomic dataset. Spatial transcriptomic data are then converted into SpatialRNA constructs within the RCTD framework, facilitating the extrapolation of gene expression landscapes and cellular proportions in spatial contexts using the least squares method. The outputs of the RCTD deconvolution analysis lay the groundwork for investigating spatial cellular dynamics through the “mistyR” R package. This toolset explores the spatial relationships of cellular assemblies within tissues, hypothesizing potential cell-to-cell interactions based on established gene expression patterns and the spatial distribution of various cellular entities.

### 2.5 HCC macrophage glycosyltransferase key genes combined with bulk data for prognostic and immunotherapy analysis

We examined the potential prognostic significance of the glycosyltransferase gene MGAT1 using data from TCGA. The “survival” R package was employed to construct a survival function, with Kaplan-Meier curves visualized via the “Survival” R package (55). To evaluate MGAT1’s impact on immunotherapy efficacy, we utilized the TIDE platform—a computational tool that leverages TCGA data to assess immune evasion mechanisms. TIDE identifies two primary modes of immune evasion: immune dysfunction and immune rejection, providing prognostic insights into patient responses to immune checkpoint inhibitors targeting PD-1, PD-L1, and CTLA-4. This analysis enhanced our comprehension of the relationship between MGAT1 expression and the effectiveness of immunotherapeutic treatments.

### 2.6 Differential analysis and prognosis of MGAT1 gene between tumor and normal tissues

To elucidate the differential expression and prognostic value of the glycosyltransferase-associated gene MGAT1 in cancerous versus healthy tissues, we conducted comprehensive analyses using TCGA data. The “Limma” R package was utilized to determine the differential expression of MGAT1 between normal and tumor cohorts (56). Survival outcomes were visualized with Kaplan-Meier curves, generated using the “Survival” R package. We developed a nomogram that integrates MGAT1 expression with clinical metadata, using the “dplyr” R package, to offer a detailed perspective on patient prognosis. To evaluate the utility of the nomogram, we performed decision curve analyses (DCA) for 1, 3, and 5-year survival intervals using the “DCA” R package. Additionally, the predictive performance of MGAT1 was assessed through ROC curve analyses, plotted with the “pROC” R package. For visual confirmation, immunohistochemical images contrasting hepatocellular carcinoma with normal tissues were obtained from The Human Protein Atlas (https://www.proteinatlas.org/).

### 2.7 Cell culture and transient transfection

Human hepatocellular carcinoma cell lines HepG2 and Huh7, along with the non-transformed epithelial cell line HL7702, were cultured in Dulbecco’s modified Eagle’s medium (DMEM, GIBCO) supplemented with 10% fetal bovine serum (FBS; Hyclone) and 100 U/L penicillin and 100 mg/L streptomycin (Thermo Fisher). The cells were maintained at 37°C in a 5% CO2 environment. For transient transfection studies, Lipofectamine 3000 (Invitrogen, Carlsbad, CA, USA) was used following the manufacturer’s protocol to introduce Negative Control (NC) and MGAT1 siRNA constructs (RiboBio, Guangzhou, China) into the HCC cells.

### 2.8 qRT-PCR

RNA was extracted from samples using the RNA Eazy Fast Tissue/Cell Kit (TIANGEN Biotech Co., Beijing), adhering to the supplier’s guidelines. cDNA synthesis was performed using the FastKing RT Kit (TIANGEN Biotech Co., Beijing) as per the provided instructions (57). Quantitative PCR assays were conducted using the SuperReal PreMix Plus (TIANGEN Biotech Co., Beijing) on the StepOnePlus Real-Time PCR System. The amplification protocol included an initial denaturation at 95°C for 15 minutes, followed by 40 cycles of 95°C for 10 seconds, 72°C for 20 seconds, and 60°C for 20 seconds.

### 2.9 CCK-8 assay

Cell viability was assessed using the Cell Counting Kit-8 (CCK-8) assay (58). Cells were seeded at a density of 1500 cells per well in 200 µl of complete growth medium in 96-well plates and incubated at 37°C. After specific experimental treatments, 20 µl of CCK-8 solution (Beyotime, Shanghai, China) was added to each well. Following a 2-hour incubation, optical density at 450nm (OD450nm) was measured using a microplate spectrophotometer.

### 2.10 Transwell assay

To evaluate cellular invasion and migration, 1×10^5 cells were seeded into transwell inserts, with Matrigel-coating (BD Biosciences, San Jose, CA) used for the invasion assay and uncoated inserts for the migration assay. The upper chamber contained serum-free medium, while the lower chamber was filled with complete DMEM. After a 24-hour incubation, cells that had migrated through the membrane were fixed with 4% paraformaldehyde and stained with 0.1% crystal violet (59). Cells that had traversed the membrane were counted under a light microscope (Thermo Fisher, Waltham, MA, USA).

### 2.11 Statistical analysis

Statistical analyses were conducted using R (version 4.3.0) with pertinent libraries, complemented by Python’s PyCharm integrated development environment. Continuous variables between paired groups were compared using the nonparametric Wilcoxon rank sum test. Spearman’s correlation coefficients were employed to assess correlations. A significance threshold of P<0.05 was applied across all analyses. Data from the CCK-8 assay were analyzed utilizing GraphPad Prism Software (version 8.3.0). Results were presented as means ± standard deviation (SD) from three independent experiments and analyzed using the Student’s t-test, with p-values <0.05 considered statistically significant.

## 3 Results

### 3.1 Analysis of HCC single-cell sequencing results

**Figure 1:**
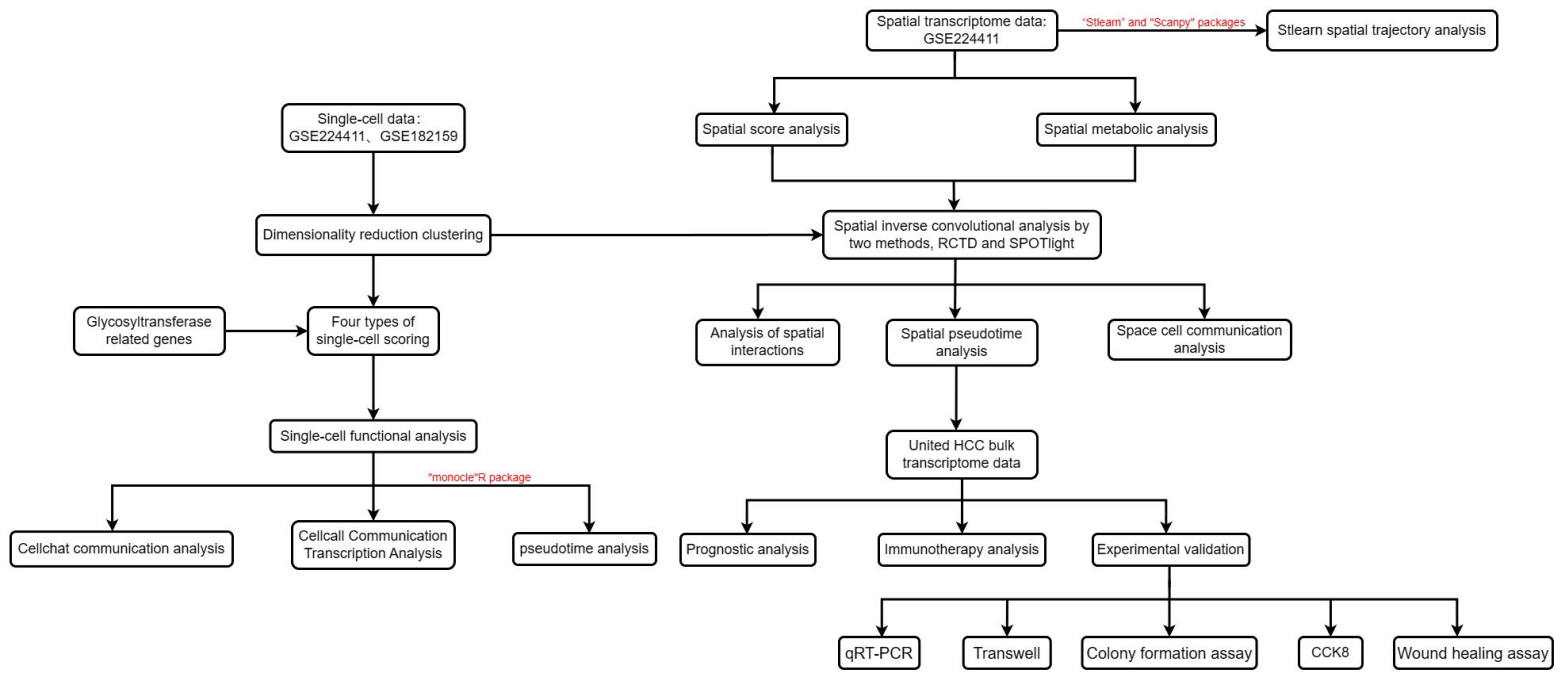
Flow chart of this study.

**Figure 2:**
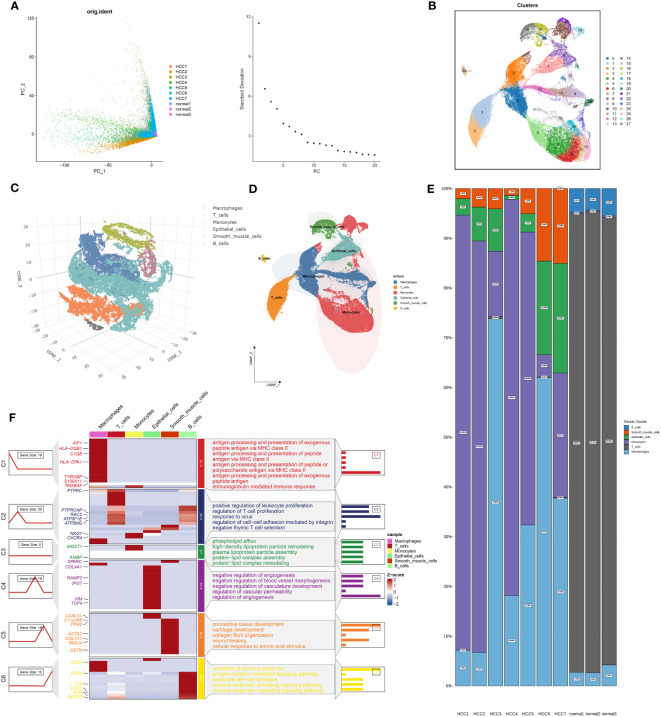
Integration of Single-Cell Sequencing and Glycosyltransferase Gene Set Filtering for Identification of HCC-Related Feature Genes. **(A)** Gene filtering of the single-cell expression matrix is depicted in the left panel, accompanied by linear dimensionality reduction through PCA clustering of the samples. The distribution of the top 20 ranked principal components (PCs) is illustrated in the right panel. **(B)** Non-linear dimensionality reduction utilizing UMAP yields a clustering of all single-cell data into 28 distinct cell clusters. **(C)** Moving forward, we meticulously conducted manual annotation of these 28 cell clusters, initially through dimensionality reduction clustering employing tSNE, resulting in the categorization of these subpopulations into six distinct cell types, elegantly portrayed within a 3D spatial context. **(D)** This manual annotation process was further extended through dimensionality reduction clustering employing UMAP, culminating in the categorization of these 28 cell subpopulations into the same six cell types. **(E)** In parallel, we scrutinized the distribution of cell proportions across the six identified cell types in diverse samples. **(F)** Subsequently, we subjected the six cell types obtained from annotation to enrichment analysis, where the leftmost line graphs delineate the transition from macrophages to B_cells in a top-to-bottom arrangement. Simultaneously, the accompanying heatmaps provided insights into gene expression patterns relative to each cell type, while the rightmost section illuminated the functional pathways enriched in each cell type, inclusive of their corresponding highly expressed genes.

**Figure 1** outlines the structured workflow of our study. We utilized single-cell data from seven HCC samples (GSE224411 and GSE182159 repositories) and compared them with three normal tissue samples. This foundational dataset facilitated the exploration of the differences between tumorigenic and non-tumorigenic cells, offering insights into the complex heterogeneity of HCC. Following rigorous quality control to exclude underperforming cells, we normalized the data and addressed batch effects, ultimately curating the top 2000 variably expressed genes for detailed analysis. Using Principal Component Analysis (PCA), we identified the ten most significant principal components based on P-value thresholds (**Figure 2A**). Employing the UMAP technique, we revealed 28 unique cellular clusters from a total of 62,511 cells (**Figure 2B**). These clusters were manually annotated, resulting in the identification of six distinct cellular phenotypes, visualized through tSNE dimensional reduction (**Figure 2C**). The integration of manual annotations with dimensional clustering allowed for precise cell type delineation and spatial representation (**Figure 2D**). Our analysis highlighted a shift in cellular composition, with a noticeable decrease in T cells and an increase in macrophages and monocytes in tumor samples compared to normal ones (**Figure 2E**). Gene ontology enrichment analysis of annotated cell types revealed key functional enrichments: macrophages were associated with immunoglobulin-mediated immune responses, T cells showed heightened viral reactivity, and both T cells and B cells exhibited robust immune activation signatures (**Figure 2F**). This comprehensive framework provides an extensive view of cellular dynamics and interactions within the HCC environment.

### 3.2 Single-cell sequencing combined with glycosyltransferase gene set screening for HCC-related feature genes

**Figure 3:**
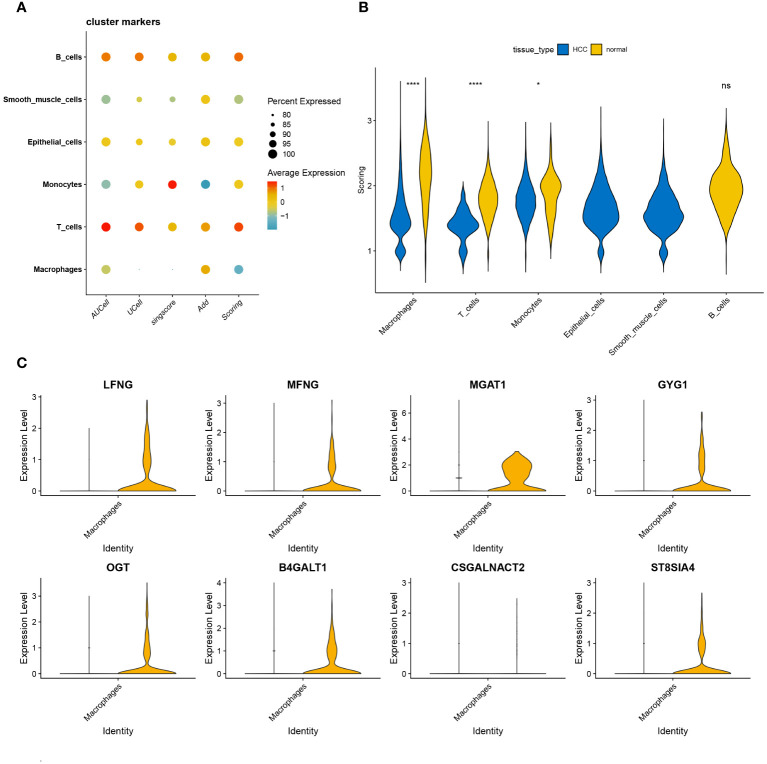
**(A)** Correlation analysis is visually represented through bubble plots. These plots exhibit the correlations, both positive and negative, between the four single-cell scoring methods and overall scores with different cell types. Bubble size corresponds to the strength of correlation, transitioning from blue (negative) to red (positive). **(B)** Glycosyltransferase-related genes are introduced, showcasing their expression disparities across different cell types within the HCC and normal groups. The HCC group is denoted in blue, while the normal group is depicted in yellow. **(C)** Violin plot of glycosyltransferase-related genes highlights their significant expression differences in macrophages between the HCC and normal groups. *p < 0.05, ****p < 0.0001. ns, no significance.

We utilized four prominent algorithms—AUCell, UCell, singscore, and AddModuleScore—to score the single-cell dataset based on a curated panel of 185 glycosyltransferase-associated genes. Integrating insights from these algorithms, we identified intriguing patterns, particularly in macrophages, which showed a significant reduction in glycosyltransferase gene transcriptional activity within HCC (**Figure 3A**). This integration illuminated distinct expression landscapes, depicted through violin plots that captured varying transcriptional footprints between HCC and normal samples. Notably, macrophages, T cells, and monocytes displayed significant transcriptional differences between HCC and normal samples, with a pervasive reduction in transcriptional activity across HCC subsets compared to normal counterparts (**Figure 3B**). Focusing on macrophages, we identified key glycosyltransferase-related genes, with seven genes prominently expressed in normal macrophages. Among these, MGAT1 emerged as the most significantly modulated gene (**Figure 3C**). Based on these findings, subsequent investigations will prioritize MGAT1, which showed a marked decrease in expression in HCC-associated macrophages.

### 3.3 CellChat dissects cellular communication in the tumor immune microenvironment

**Figure 4:**
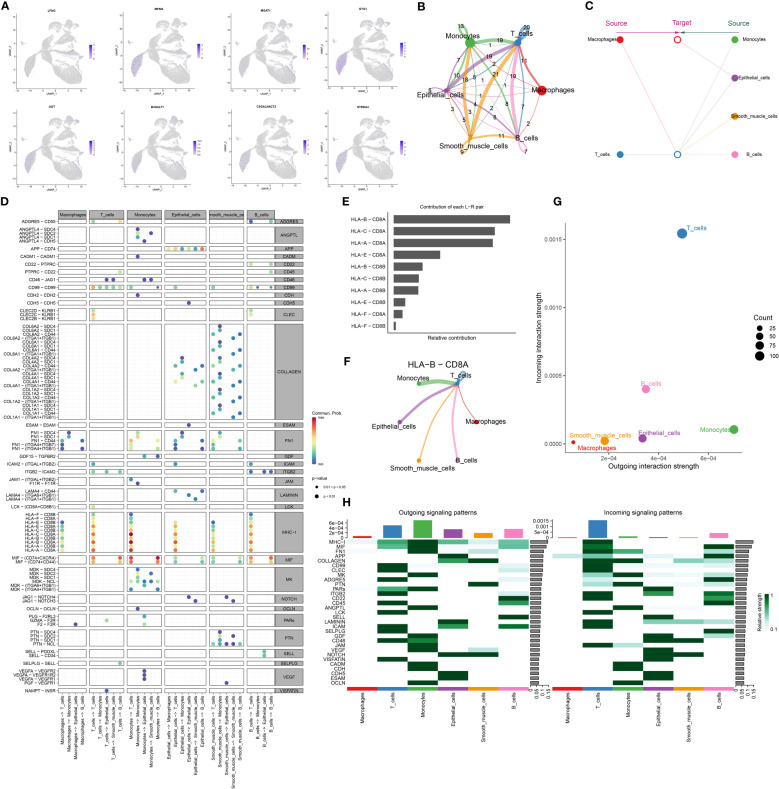
HCC cellchat cell communication analysis. **(A)** Visualization of significantly differentially expressed glycosyltransferase-related genes in macrophages in UMAP nonlinear downward clustering, with darker colors representing higher expression of the gene within the region. **(B)** Cell-cell interactions. The thickness of the interconnecting lines and the numbers on them represent the number of interactions between cells. **(C)** Hierarchical diagram showing the hierarchical relationship of the six cell types in cellular communication. **(D)** Dot plot showing ligand-receptor pairs that are prominent in cellular communication; ligand-receptor pairs are categorized according to ligand-receptor family pairs. **(E)** Histogram of the contribution of each ligand-receptor pair. **(F)** Chord diagrams of HLA-B-CD8A intercellular communication in six cell types. **(G)** Dot plots of input and output intensities in intercellular interactions in six cell types. **(H)** Heatmap showing the overall outward and inward signaling patterns of the six cell types.

Utilizing UMAP analysis, we quantified the expression of glycosyltransferase-associated signature genes, confirming that none were notably overexpressed in macrophages, consistent with our initial observations (**Figure 4A**). Further, we mapped the cellular communication landscape, visualizing interaction magnitudes and affiliations among cellular clusters (**Figure 4B**). This analysis highlighted the central role of macrophages in interactions with various cellular counterparts (**Figure 4C**). To gain insights into intercellular communication and ligand-receptor dynamics, we refined our single-cell data analysis. A key observation was the prominent association of MHC-I with cellular clusters (**Figure 4D**). Additionally, MHC-I’s influence on signaling pathways was underscored by the HLA-B-CD8A interaction, shaping our future research direction (**Figure 4E**). The relational matrix of HLA-B-CD8A among cellular clusters revealed a unique affinity between T cells and this ligand-receptor pair within the macrophage context (**Figure 4F**). Evaluating the ingress and egress interaction intensities for HLA-B-CD8A across cellular groups, T cells exhibited the highest combined association (**Figure 4G**). Heatmaps depicting signaling directionality further revealed intricate interactions among cellular groups, with a notable link consistently observed between T cells and MHC-I (**Figure 4H**). Our forthcoming research will focus on deciphering the interactions between macrophage-derived HLA-B ligands and CD8A receptors on T cells in the progression of HCC.

### 3.4 Pseudotime analysis and intercellular communication analysis

**Figure 5:**
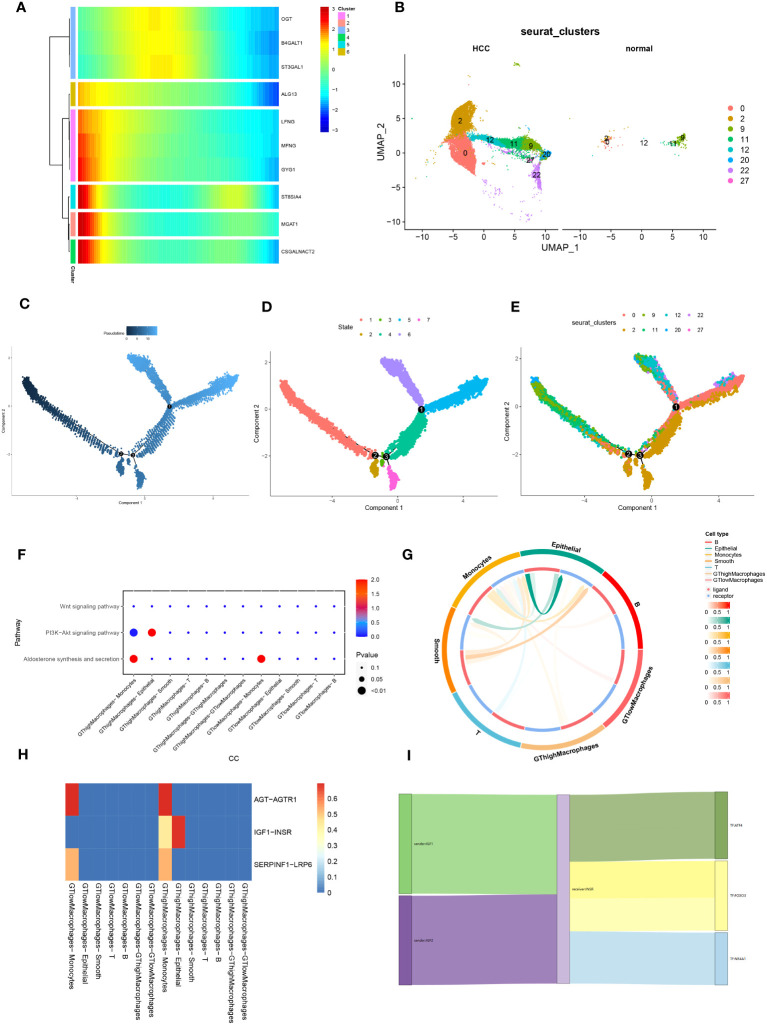
Pseudotime analysis and cell-to-cell communication analysis. **(A)** Developmental heatmap of macrophage glycosyltransferase-related genes, from left to right indicates time progression, while the color from blue to red indicates low to high expression. **(B)** Comparative Figure of UMAP nonlinear descending clustering of macrophages between HCC and normal groups. **(C)** Figure of macrophage pseudotime, and the color from black to blue indicates the progression of time. **(D)** Developmental Figure of cell trajectories of tumor macrophages, which in combination with A and C can observe the developmental trajectories of different macrophage populations in tumors. **(E)** Developmental Figure of macrophage trajectories in normal and tumor groups, combined with B and C to observe the developmental trajectories of different macrophage populations in normal and tumor groups. **(F)** Bubble plots of the correlation of 30 metabolic pathways between different cell types and macrophages with high/low expression of glycosyltransferase-related genes; the size of the bubbles represents the size of the correlation, whereas the color from blue to red indicates from negative to positive correlation. **(G)** Correlation circle plots between different cell types. **(H)** Heatmap of cell-cell ligand-receptor correlation. **(I)** Ligand-receptor-transcription factor Sankey Figure between macrophages and epithelial cells with high expression of glycosyltransferase-related genes.

To delineate cellular differentiation and evolutionary trajectories at the single-cell level, we utilized pseudotime and cell trajectory analyses, constructing developmental pathways for cells. Heatmaps were employed to depict glycosyltransferase-associated gene expression across developmental stages, revealing elevated MGAT1 expression in early tumorigenic phases (**Figure 5A**). Analysis of macrophage gene expression in HCC versus normal samples highlighted significant disparities in glycosyltransferase-linked genes (**Figure 5B**). Using the “monocle” R package, we traced the spatial-temporal evolution of macrophages, observing a systematic increase in the pseudotime continuum from initial to terminal phases (**Figure 5C**). Integrating this with **Figure 5A**, we identified seven cellular subgroups, each representing unique transitional states in macrophage maturation. Notably, clusters 1, 3, and 5, corresponding to early, intermediate, and advanced cellular phases, exhibited distinct evolutionary paths with increasing pseudotime (**Figure 5D**). Further examination revealed a transition: clusters dominant in normal samples (clusters 5 and 9) shifted to those prevalent in HCC (clusters 0 and 2) as pseudotime advanced (**Figure 5E**). These findings underscore a systematic progression from normal to tumorigenic clusters, as reflected by glycosyltransferase-centric genes in pseudotime and trajectory analyses.

Exploring macrophage interactions with various cell types, we categorized them based on glycosyltransferase gene expression into high (GThigh) and low (GTlow) subsets. Pathway enrichment analysis in GThighMacrophages and epithelial cells predominantly highlighted the oncogenesis-associated PI3K-Akt signaling pathway (**Figure 5F**). Circle plots illustrated the ligand-receptor interactions and their magnitudes across cellular phenotypes (**Figure 5G**). Comparing ligand-receptor interaction heatmaps between GTlowMacrophages and other cells with GThighMacrophages and their counterparts revealed a distinct IGF1-INSR interaction specifically in GThighMacrophages versus epithelial cells (**Figure 5H**). Building on this, we constructed Sankey diagrams to encapsulate ligand-receptor-transcription factor networks, highlighting the crosstalk between GThighMacrophages and epithelial cells (**Figure 5I**).

### 3.5 Spatial transcriptome sequencing unites macrophage glycosyltransferase feature genes

**Figure 6:**
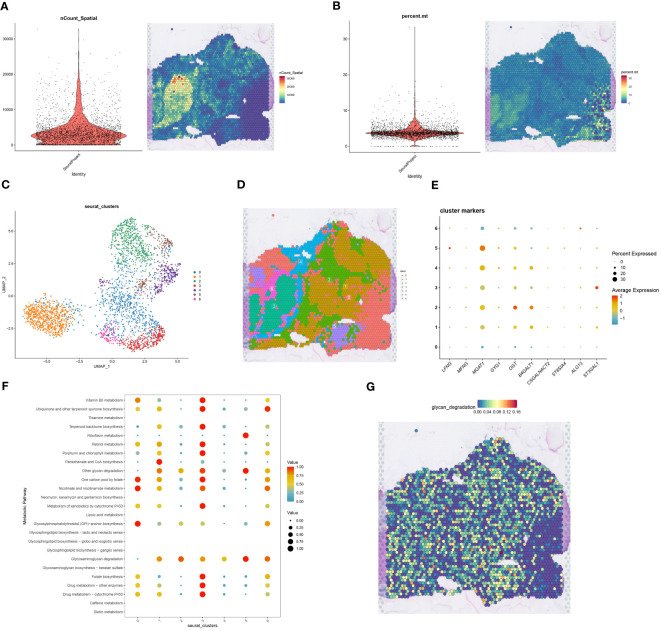
Spatial transcriptome sequencing associates Macrophages-related glycosyltransferase genes. **(A)** The left Figure shows a violin plot of cell number in spatial transcriptome data after QC, and the right Figure visualizes cell number on tissue sections of spatial transcriptome after QC, with blue to red indicating a gradual increase in cell number. **(B)** The left Figure shows a violin plot of mitochondrial genes in the spatial transcriptome data, and the right Figure visualizes the expression of mitochondrial genes on spatial transcriptome tissue sections, with blue to red indicating low to high gene expression. **(C)** The spatial transcriptome data were classified into seven cell clusters after UMAP nonlinear dimensionality reduction clustering. **(D)** Projections of the seven cell clusters after dimensionality reduction clustering on spatial transcriptome tissue sections. **(E)** Bubble plots of correlation of macrophage glycosyltransferase-related gene expression in the 7 cell populations, the size of the bubbles represents the correlation size, while the color from blue to red indicates the correlation from negative to positive correlation. **(F)** Correlation bubble plots between 7 cell populations and 30 metabolic pathways. **(G)** Visual expression Figure of glycan_degradation metabolic pathways selected from F in spatial transcriptome tissue sections.

We obtained spatial transcriptome sequencing data specific to an HCC patient from the GSE224411 dataset within the GEO database. Ensuring data integrity, rigorous quality control measures were applied to this spatial transcriptome dataset. This scrutiny provided spatial insights into both cellular distribution (**Figure 6A**) and mitochondrial gene dispersion (**Figure 6B**). Post-quality control, the data underwent cleansing to exclude mitochondrial and ribosomal genes, followed by normalization and depth correction via the SCTransform methodology. This led to the identification of seven spatially distinct cell clusters, visualized through UMAP projections (**Figures 6C, D**). Integrating HCC macrophage-centric glycosyltransferase feature genes from single-cell analysis with these spatial clusters culminated in a clear bubble map representation (**Figure 6E**). To investigate metabolic dynamics across cell clusters, we used the “scMetabolism” R package (**Figure 6F**), focusing on glycan degradation due to its pivotal role in tumor progression, immune evasion, and cellular signaling. This analysis resulted in a spatial representation of glycan degradation activity (**Figure 6G**). Given the low expression of glycosyltransferase-associated genes in HCC and the prominent glycan degradation functionality in cell cluster 5, we inferred that cell cluster 5 likely represents normal tissue.

### 3.6 RCTD deconvolution analysis and spatial cell development analysis

**Figure 7:**
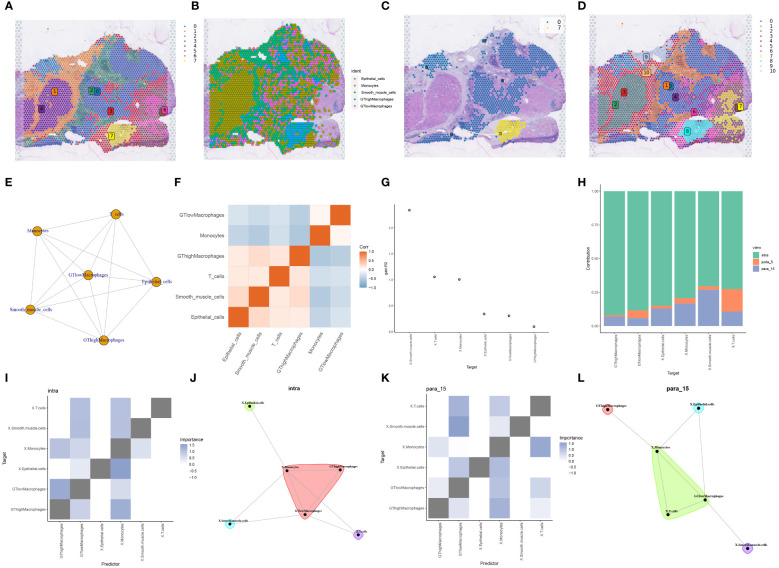
RCTD deconvolution analysis with spatial cell development analysis. **(A)** Louvain clustering divides the spatial transcriptome data into 8 cell clusters. **(B)** Spatial transcriptome tissue sections presenting cell types in single cell data deconvoluted to cell types in spatial transcriptome data by RCTD deconvolution method. **(C)** Development and transfer of cluster 0 to cluster 7 in spatial transcriptome tissue sections. **(D)** Leiden clustering divides the spatial transcriptome data into 11 cell clusters. **(E)** Correlation network diagram between different cell types. **(F)** Heatmap of correlation between different cell types. **(G)** The custom R package “mistyR” analyzes the improvement stats of different cell types and displays their correlation sizes. **(H)** Histogram of the contribution of the three customized functions intra, juxta_5 and para_15 to the cellular importance measure. **(I)** Heatmap of correlations between different cell types computed by intra. **(J)** Correlations between different cell types calculated by intra. **(K)** Heatmap of correlations between different cell types calculated by para_15. **(L)** Correlations between different types of cells calculated by para_15.

To bridge the resolution gap between spatial transcriptome technologies and single-cell sequencing, we employed the RCTD deconvolution technique alongside single-cell data to deduce cell type fractions within heterogeneous samples. The meticulous RCTD deconvolution identified two macrophage classifications, GThighMacrophages and GTlowMacrophages, based on glycosyltransferase gene expression. Utilizing the “Stlearn” and “Scanpy” Python packages, the spatial transcriptome data underwent clustering and normalization, revealing eight distinct cell clusters (**Figure 7A**). On a broader scale, GTlowMacrophages were predominantly found in cell cluster 0, while GThighMacrophages were mainly in cell cluster 7 (**Figure 7B**). Tracing the cell developmental trajectory spatially, a clear shift from cell cluster 0 to cell cluster 7 within tumor regions was evident (**Figure 7C**). Delving deeper into spatial cellular delineations, Leiden clustering was used, resulting in 11 intricate cell clusters (**Figure 7D**). The intercellular network topology illustrated the complex web of cellular interactions (**Figure 7E**). Notably, an intercellular correlation intensity heatmap revealed an inverse relationship between GTlowMacrophages and T cells, suggesting reduced T cell expression in HCC samples, potentially influencing HCC progression (**Figure 7F**). Utilizing the “mistyR” R toolkit, we explored spatial cell-cell interactions, scrutinizing enhancement metrics across diverse cell types (**Figure 7G**). Further insights into contributions, interactions, and cell-type correlations were obtained through the intra, juxta_5, and para_15 functionalities within “mistyR” (**Figure 7H**). Contribution histograms vividly articulated correlation magnitudes and interdependencies across cell types, highlighting the significance of intra and para_15 (**Figures 7I–L**).

### 3.7 SPOTlight deconvolution analysis with spatial pseudotime analysis

**Figure 8:**
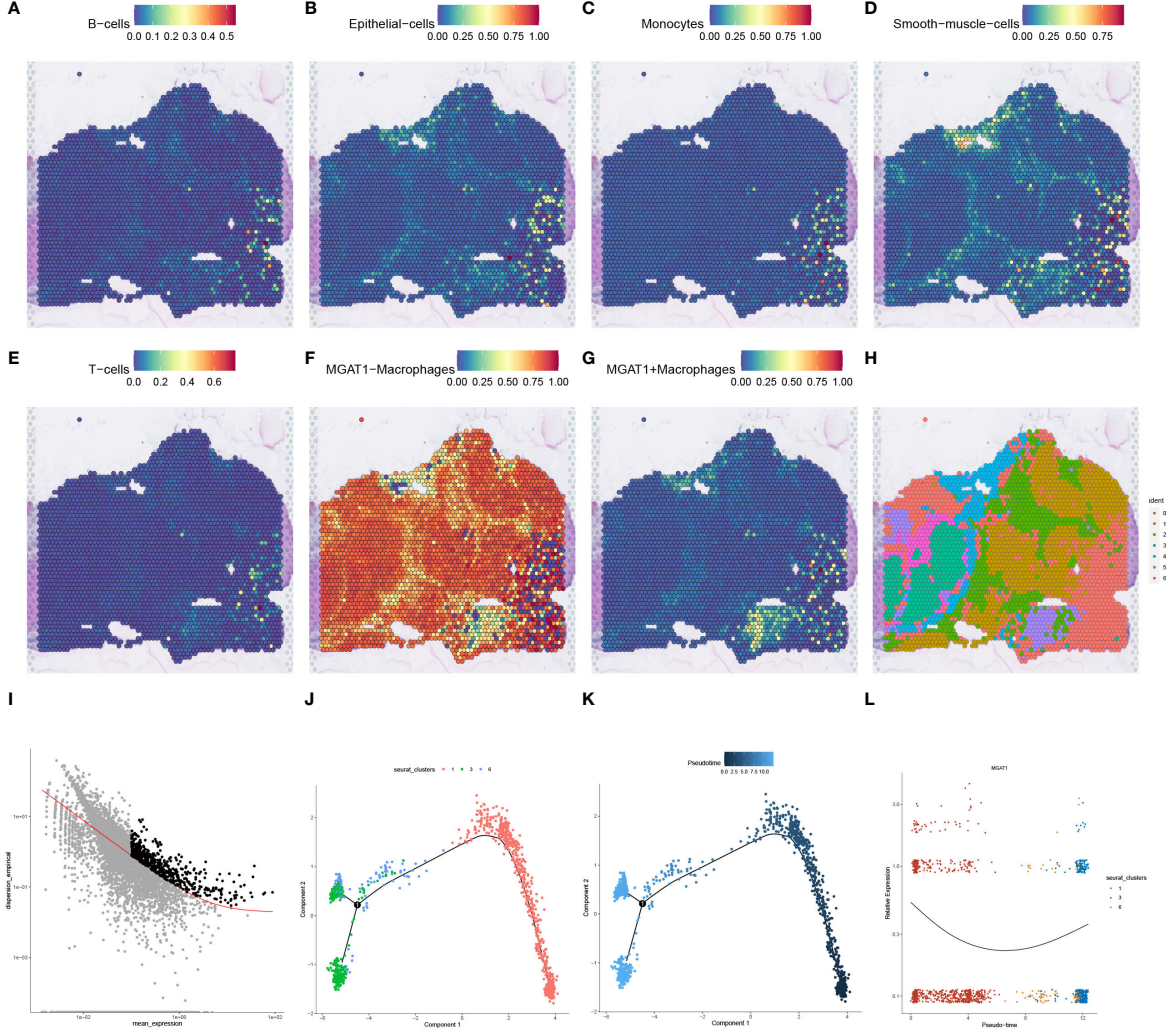
SPOTlight deconvolution analysis with spatial pseudotime analysis. **(A–G)** Expression of different cell types in single-cell data visualized by SPOTlight deconvolution on spatial transcriptome tissue sections. **(H)** louvain clustering of seven cell populations on spatial transcriptome tissue sections for presentation. **(I)** Cell trajectory analysis of macrophage monocytes shows different cell clusters with distinct cell lineages. **(J)** Cell development trajectories of three populations of cells highly expressing MGAT1-negative macrophages. **(K)** Pseudotime course of three populations of cells with high expression of MGAT1 macrophages, with gradual progression in time indicated from black to blue. **(L)** Changes in MGAT1 gene expression in cluster 1, cluster 3, and cluster 6 as the pseudotime progresses.

To map the spatial expression trajectories of various cellular entities, we applied the SPOTlight deconvolution technique, correlating annotated single-cell data with spatial datasets. This analysis illuminated the expression dynamics across different cell types, including B-cells, epithelial cells, monocytes, smooth muscle cells, T-cells, MGAT1-Macrophages, and MGAT1+Macrophages (**Figures 8A–G**). Through dimensionality reduction clustering, we identified seven distinct spatial cell conglomerates (**Figure 8H**). Insights from **Figures 8A–H** revealed that MGAT1-Macrophages exhibited significantly elevated expression within the tumor environment, corroborating our earlier single-cell analysis highlighting MGAT1’s role in HCC macrophages.

Proceeding to cell trajectory analysis using the discriminant downscaling tree (**Figure 8I**), we observed that clusters with high MGAT1-Macrophage expression (Clusters 1, 3, and 6) underwent a transformative journey in pseudotime. As pseudotime progressed, cluster 1 bifurcated into clusters 3 and 6 at node 1 (**Figures 8J, K**). Notably, examining the pseudotime dynamics of the MGAT1 gene alone revealed a marked decrease in MGAT1 expression as cluster 1 transitioned into cluster 6, and further diminished expression from cluster 6 to cluster 3 (**Figure 8L**). These findings suggest that cluster 1 represents the primary tumor state, which evolves into clusters 3 and 6 over time, potentially driving tumor progression.

### 3.8 Spatial cell-talk analysis

**Figure 9:**
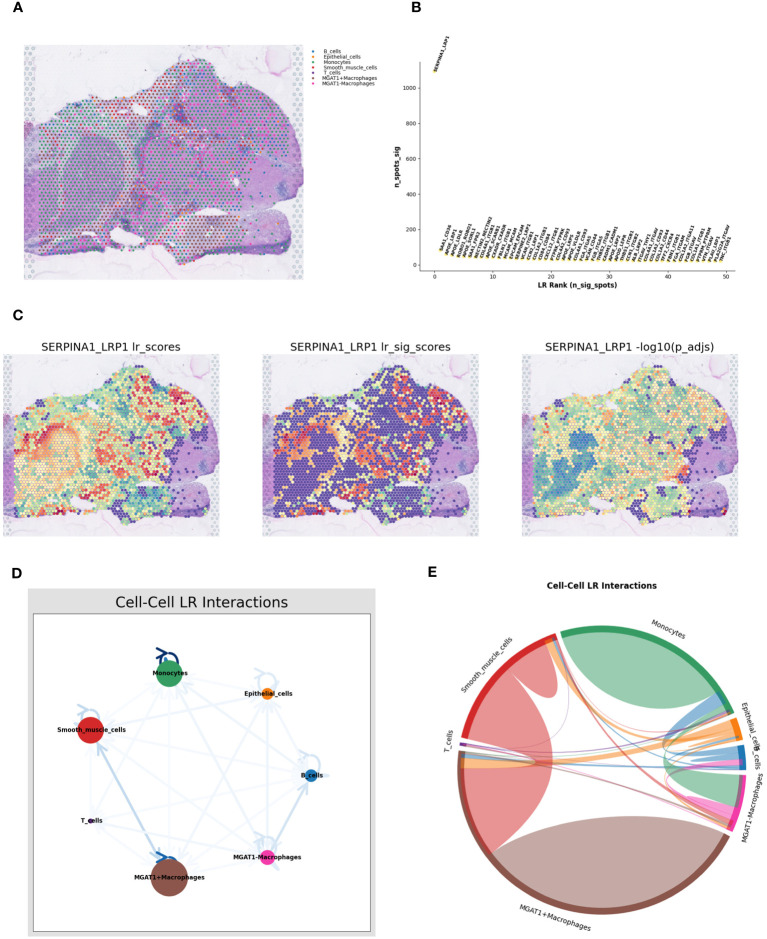
Spatial cell-talk analysis. **(A)** Macrophages fused with the MGAT1 gene and other cell types were visualized by deconvolution from single-cell data integrated into spatial transcriptome data. **(B)** Top 50 ligand-receptor pairs among MGAT1 macrophages. **(C)** Expression of the top-ranked ligandreceptor pair SERPINA1_LRP1 in spatial transcriptome tissue sections in **(B)**. **(D)** Network Figure of cell-cell ligand-receptor interactions. **(E)** Circle diagram of cell-cell interactions.

Using the RCTD deconvolution strategy, we integrated single-cell datasets with spatial transcriptomic data, achieving precise deconvolution of MGAT1+Macrophages, MGAT1-Macrophages, and other annotated cellular entities (**Figure 9A**). Investigating ligand-receptor interactions, we utilized the “Stlearn” toolkit in Python, revealing the top 50 ligand-receptor pairs, each highlighted with a spatial score (**Figure 9B**). The SERPINA1_LRP1 pair emerged as the most significant interaction within the spatial context, prompting a detailed exploration of spatial cellular dialogues. Spatial enrichment analyses focused on the SERPINA1_LRP1 pair revealed a strong concentration within the tumor area (**Figure 9C**). Focusing on the reduced MGAT1 expression in tumor regions, we analyzed the interaction between MGAT1-Macrophages and monocytes, mediated by the SERPINA1_LRP1 ligand-receptor pair (**Figures 9D, E**). This detailed analysis unravels the complex spatial cellular interactions within the tumor microenvironment, highlighting the potential role of MGAT1-Macrophages and monocytes in the context of HCC.

### 3.9 MGAT1+Macrophages combined with bulk data for prognostic and immunotherapeutic target analysis

**Figure 10:**
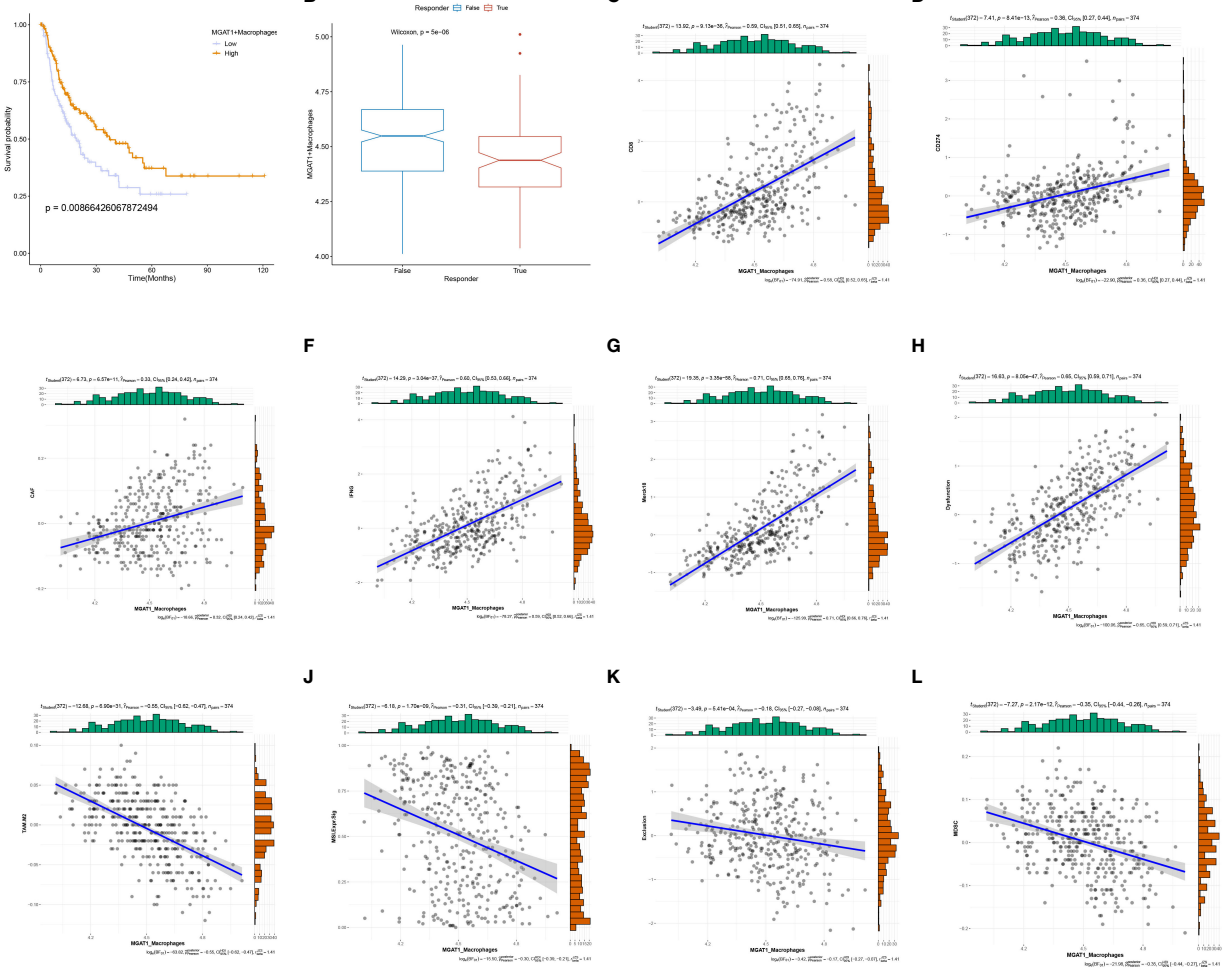
MGAT1-based prognosis with immunotherapy target analysis. **(A)** K-M curves showing the change in survival likelihood over time progression between high/low expressing MGAT1 positive macrophages. **(B)** Box line plots of immunotherapy for the determination of therapeutic efficacy for MGAT1-positive macrophages with different levels of expression. **(C–L)** Scatter plots of correlations between MGAT1-positive macrophages and corresponding immune targets, functions, or drugs.

To assess the prognostic significance of MGAT1+Macrophages in HCC, we stratified patients into high and low expression cohorts based on an optimal threshold (P<0.05). Kaplan-Meier curves indicated that the low MGAT1+Macrophage expression cohort had poorer survival outcomes compared to the high-expression cohort (**Figure 10A**). This finding aligns with our previous observations, suggesting that low MGAT1 expression in HCC correlates with poor prognosis. Interestingly, a notable difference in TIDE scores was observed between the low and high MGAT1+Macrophage expression groups, indicating that lower levels of MGAT1+Macrophages might predict better responses to immunotherapy (**Figure 10B**). Correlation matrices showed positive associations between MGAT1+Macrophages and markers such as CD8, CD274, CAF, IFNG, Merck18, and Dysfunction (**Figures 10C–H**). Conversely, negative associations were found with markers such as TAM.M2, MSI.Expr.Sig, Exclusion, and MDSC (**Figures 10I–L**). These results underscore the critical role of MGAT1+Macrophages in the tumor microenvironment, elucidating their potential impact on HCC prognosis and their role in modulating immunotherapeutic responses.

### 3.10 Differences in MGAT1 expression between tumor samples and normal samples

**Figure 11:**
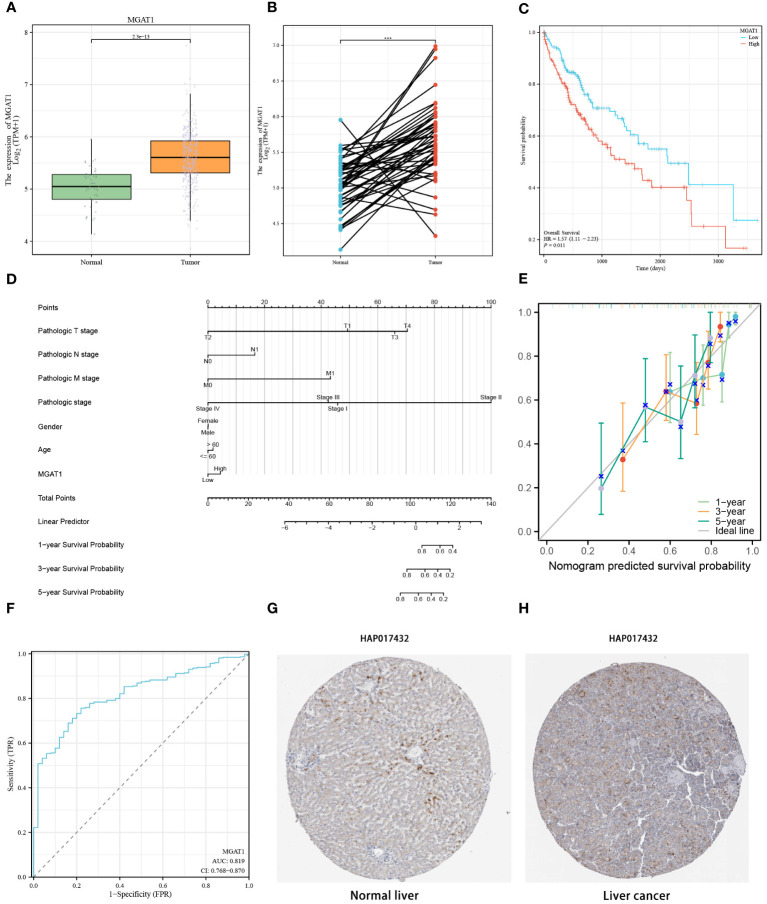
MGAT1 Gene Expression in Tumor and Normal Samples. **(A)** Differential MGAT1 Gene Expression: A box line plot is presented, elucidating the variance in MGAT1 gene expression between samples derived from normal and HCC tissues. **(B)** Differential expression of MGAT1 gene in paired tumor samples and normal samples. **(C)** Survival Analysis: Kaplan-Meier curves elegantly portray the dynamic shift in survival probabilities among HCC patients based on their MGAT1 expression levels. The curves discernibly contrast the outcomes for patients exhibiting high versus low MGAT1 expression as time progresses. **(D)** The nomogram constructed by combining MGAT1 gene and multiple clinical information. **(E)** Decision calibration curves at 1, 3 and 5 years. **(F)** Diagnostic ROC curves for the MGAT1 gene. **(G)** Immunohistochemical Profiling - Normal Liver Tissues: Immunohistochemical results thoughtfully illustrate the presence and localization of the MGAT1 gene product in tissues from healthy liver specimens. **(H)** Immunohistochemical Profiling – Liver Cancer Tissues: Immunohistochemical findings provide insights into the distribution and intensity of the MGAT1 gene product within tissues afflicted by liver cancer. ***p < 0.001.

To delve deeper into the disparities in MGAT1 gene expression between tumor and normal tissues, we analyzed TCGA bulk datasets for enhanced visualization. This analysis revealed a significant increase in MGAT1 expression in tumor samples compared to normal tissues (**Figure 11A**). A similar pattern was observed in paired sample comparisons, where tumor specimens consistently exhibited higher MGAT1 expression than their normal counterparts (**Figure 11B**). Kaplan-Meier plots indicated that lower MGAT1 expression was associated with better survival outcomes, contrasting sharply with those exhibiting higher expression (**Figure 11C**). By integrating MGAT1 expression profiles with various clinical metrics, we developed a precision-guided nomogram (**Figure 11D**), whose predictive accuracy was validated by well-calibrated decision curves at 1, 3, and 5-year intervals (**Figure 11E**). The diagnostic capability of MGAT1 was further corroborated by a receiver operating characteristic (ROC) curve, which demonstrated an impressive AUC of 0.819 (**Figure 11F**). Immunohistochemical assays comparing healthy liver sections with hepatocarcinoma tissues highlighted the elevated presence of MGAT1 in tumor matrices (**Figures 11G, H**). These findings collectively underscore the pivotal role of MGAT1 in hepatocarcinoma, emphasizing its importance as a diagnostic and prognostic marker.

### 3.11 Analysis of MGAT1 immune infiltration and immunotherapy

**Figure 12:**
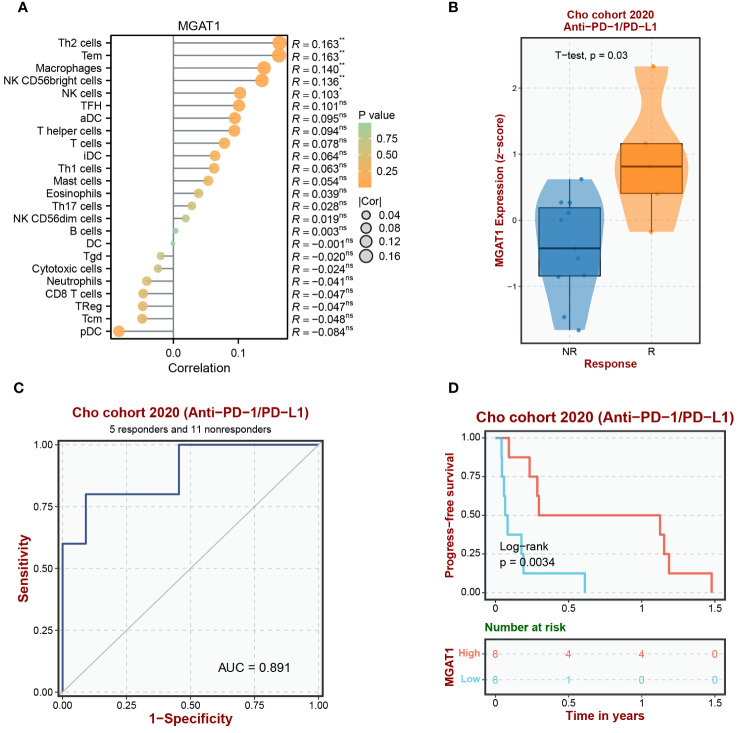
Analysis of MGAT1 immune infiltration and immunotherapy. **(A)** It begins by portraying immune infiltration patterns driven by the MGAT1 gene through a lollipop figure. **(B)** Subsequently, the introduction of an external cohort to investigate the Anti-PD-1/PD-L1 immune response associated with MGAT1 is presented via a box-and-line plot. **(C)** Further, the figure delves into the evaluation of MGAT1’s predictive capacity as a biomarker for discerning Anti-PD-1/PD-L1 responsiveness across diverse external cohorts, effectively utilizing ROC curves. **(D)** Lastly, the analysis extends to explore Progression-Free Survival (PFS) in the context of Anti-PD-1/PD-L1 therapy, unveiling the influence of MGAT1 gene expression on patient outcomes within an external cohort. Collectively, these integrated visualizations underscore MGAT1’s multifaceted role in shaping immune responses and its consequential relevance within the domain of immunotherapeutic interventions. *p < 0.05, **p < 0.01. ns, no significance.

To understand the immune landscape influenced by MGAT1 within the tumor environment and its implications for immunotherapeutic outcomes, we conducted an integrative analysis combining TCGA bulk data with supplementary datasets. Our assessment revealed a strong correlation between MGAT1 expression and macrophage infiltration within tumor samples (**Figure 12A**). Further investigation of MGAT1’s relationship with the immunotherapeutic target PD-1, using the GEO dataset GSE126044, revealed a significant association. Patients in the Cho et al. dataset with high MGAT1 expression showed increased responsiveness to Anti-PD-1/PD-L1 treatments (**Figure 12B**). Supporting MGAT1’s potential as a predictive biomarker, a ROC curve indicated a high AUC of 0.891, demonstrating its efficacy in predicting responsiveness to Anti-PD-1/PD-L1 therapies (**Figure 12C**). Additionally, a Progression-Free Survival (PFS) analysis within the Cho et al. cohort highlighted that individuals with elevated MGAT1 expression had better PFS outcomes following Anti-PD-1/PD-L1 therapy, compared to those with lower MGAT1 expression (**Figure 12D**). These findings collectively emphasize the crucial role of MGAT1 in shaping tumor-immune interactions and its influence on the effectiveness of immunotherapy.

### 3.12 Experimental verification that MGAT1 promotes proliferation and migration of hepatocellular carcinoma cells

**Figure 13:**
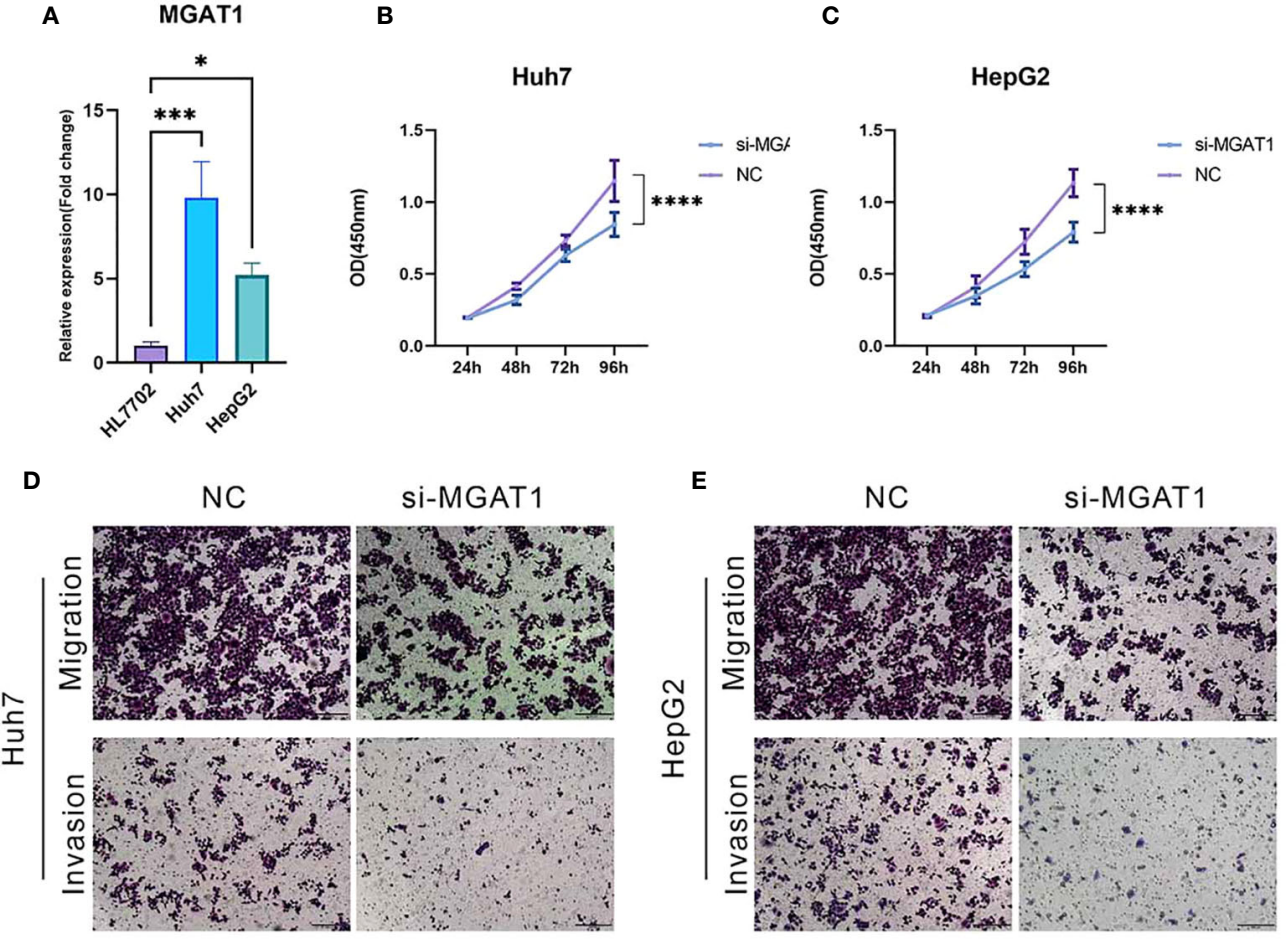
MGAT1 was shown to enhance the proliferation, invasion and migration of hepatocellular carcinoma cells. This was assessed by various assays: **(A)** qPCR assay to visualize MGAT1 expression in normal hepatocytes, HepG2 cell line and Huh7 cell line. **(B)** CCK-8 assay with untreated HepG2 cell lines and HepG2 cell lines with knockdown of MGAT1. **(C)** CCK-8 assay with untreated Huh7 cell line ands Huh7 cell line with knockdown of MGAT1. **(D)** Transwell assay, experiments were performed on untreated HepG2 cell line and HepG2 cell line knocked down MGAT1 to verify the migration and invasion power. **(E)** Transwell assay with untreated Huh7 cell line and Huh7 cell line with knockdown of MGAT1 to verify the migration and invasiveness. *p < 0.05, ***p < 0.001, ****p < 0.0001.

qPCR validation revealed a significant upregulation of MGAT1 expression in hepatocellular carcinoma cell lines HepG2 and Huh7 compared to normal hepatocytes, with statistical significance supporting this observation (**Figure 13A**). This finding is consistent with our bioinformatic analyses. To investigate the role of MGAT1 in hepatocellular carcinoma pathophysiology, we conducted a series of *in vitro* assays.

The CCK-8 assay demonstrated a significant reduction in cellular proliferation over time following MGAT1 knockdown in both HepG2 and Huh7 cell lines (**Figures 13B, C**). Additionally, transwell assays showed a marked decrease in the migratory and invasive capabilities of MGAT1-depleted hepatocellular carcinoma cells (**Figures 13D, E**). These results collectively highlight the oncogenic role of MGAT1, emphasizing its importance in promoting the proliferation, invasiveness, and motility of hepatocellular carcinoma cells.

## 4 Discussion

Glycosyltransferases execute essential cellular modifications, adjusting the glycosylation processes of proteins, lipids, and nucleic acids, thereby influencing their functional integrity and stability (60–62). These enzymes are involved in a wide range of biological activities, including cell signaling, cellular interactions, adhesion, tissue development, and cancerous transformations (63–66). Given the crucial role of oligosaccharide structures—products of glycosylation—in facilitating cellular communication and immune regulation, glycosyltransferases have become central to mechanistic disease studies and therapeutic design (67–69). Notably, reduced glycosyltransferase activity has been linked to decreased expression of intercellular adhesion molecules, leading to increased motility and invasiveness of cancer cells (70, 71). These enzymes also govern the glycosylation patterns of tumor antigens, affecting the complex interactions of tumor immune surveillance and evasion (72, 73). In tandem, these enzymes exert regulatory control over the glycosylation finesse of tumor epitopes, influencing the intricate dance of tumor immunosurveillance and evasion (74). Furthermore, the abnormal expression of glycosyltransferases can trigger a series of oncogenic signals, promoting cancer spread through epithelial-mesenchymal transition processes (24). However, the detailed functions of glycosyltransferases in the development of hepatocellular carcinoma (HCC) remain largely unexplored. Therefore, comprehensive studies focusing on their relationship with HCC are essential to uncover deeper insights into HCC pathobiology and to propose novel therapeutic strategies.

Conventional genomic and transcriptomic approaches, despite their transformative impact, have frequently struggled to accurately depict cellular diversity. These methodologies often produce ensemble reads from large cell groups, resulting in averaged transcriptional profiles that obscure individual cellular characteristics (75). The emergence of single-cell sequencing has revolutionized this field, enabling detailed analysis of cellular heterogeneity, discovery of previously unidentified cell types, tracking of cellular development, and identification of aberrant cell populations in diseases (76). Nevertheless, a significant limitation of single-cell sequencing is its lack of spatial context, leaving the spatial organization of complex HCC tissue architectures unexplored. Addressing this gap, spatial transcriptomics has been introduced, combining RNA sequencing precision with spatial resolution to create detailed transcriptional and spatial maps of tissue sections (77). By integrating the fine resolution of single-cell sequencing with the spatial insight of transcriptomic mapping, a comprehensive framework for HCC research is formed. This integrative approach allows for precise identification of cells, their spatial localization within tissues, the elucidation of intercellular interactions, and the interpretation of the spatial and temporal organization of cell populations. This holistic perspective promises to reveal cellular development, physiological coordination, disease mechanisms, and personalized therapeutic targets, paving the way for customized treatment strategies in the future.

In this investigation, we integrated single-cell sequencing and spatial transcriptomics to intricately examine the transcriptional landscape of glycosyltransferase-related loci within HCC. This multifaceted approach provided profound insights into the potential roles of these loci in tumorigenesis and immunotherapy. The data unveiled significant heterogeneity in the transcriptional profiles of glycosyltransferase-associated genes across various HCC contexts, with notable variations observed in tumor microenvironments and specific cellular niches as identified through spatial transcriptomic analysis. Importantly, single-cell profiles indicated a reduced expression of these genes within HCC-associated macrophages compared to normal macrophages, suggesting a tumor-induced disruption in glycosyltransferase activity specific to macrophages (78). This decreased expression could lead to reduced glycosylation of macrophage surface molecules, potentially altering their immunomodulatory properties and impacting their immune surveillance capabilities (79). Given the pivotal role of macrophages in shaping the tumor microenvironment (80), the altered glycosyltransferase landscape and subsequent functional reprogramming may significantly influence the initiation and progression of the HCC tumor microenvironment. It is crucial to recognize that macrophages play a key role in driving inflammatory and fibrogenic processes within HCC, thereby affecting tumor dynamics (81). Moreover, since glycosyltransferases are crucial regulators of cellular adhesion and invasion (82), and considering the inherent connection between macrophage invasiveness and their adhesive properties, a decline in glycosyltransferase expression might alter macrophage adhesion, thus impacting their tissue infiltration behavior and potentially mitigating tumor spread and metastasis.

In our comprehensive deconvolution analysis, spatial transcriptomics revealed significantly reduced expression of glycosyltransferase signature genes within the core of HCC tumor landscapes, indicating a potential decline in glycosylation fidelity. This finding led to the hypothesis that the hypoxic environment in the tumor microenvironment, resulting from unchecked proliferative rates and irregular vascular supply, may induce a phenotypic shift in macrophages from the tumoricidal M1 phenotype to the tumor-promoting M2 phenotype (83). Notably, these M2-skewed macrophages, characterized by their immunosuppressive properties and ability to promote tumor angiogenesis, may act to alleviate the hypoxic stress that contributed to their formation (84). Additionally, our analyses revealed an inverse relationship between TAM.M2 and MGAT1+ Macrophages. This hypoxic condition, known to alter both metabolic and signaling pathways in cancer cells (85), could modify the expression environment of glycosylation regulators, thus impacting the formation and functionality of glycosyltransferases. This hypothesis aligns with existing literature, which highlights the sensitivity of glycosyltransferase synthesis, structure, and function to the hypoxic conditions of the tumor microenvironment (86–88).

Employing spatial transcriptomic techniques, we probed the intricate spatial heterogeneity of glycosyltransferases within the HCC environment, integrating glycosyltransferase-focused gene expression with cellular identities and spatial architectures. Our spatial metabolic analysis of HCC samples disclosed a marked increase in glycan catabolism within tumoral regions, corroborating our previous findings of disrupted glycosylation in these areas. Notably, we observed a strong correlation between macrophages with elevated glycosyltransferase gene expression and epithelial cells, further linked to the aberrantly activated PI3K-Akt signaling pathway—a common anomaly in various cancers, including HCC (89). This upregulated PI3K-Akt activity in cancer contexts promotes cellular proliferation, inhibits apoptosis, and enhances invasive behaviors, contributing to tumor growth and spread (90). This signaling complexity is intertwined with dysregulated glycosyltransferase expression, reflecting altered glycosylation processes (91). Interestingly, this molecular cascade significantly impacts macrophage biology (92). Its abnormal activation can drive macrophages toward an M2-skewed phenotype, known for its immunosuppressive characteristics, thereby fostering an environment that supports tumor evasion and growth (93–95). Considering the epithelial origin of HCC (96), our study highlighted the crucial role of the PI3K-Akt pathway in orchestrating the oncogenic interactions between glycosyltransferase-rich macrophages and epithelial cells. In this context, we identified an IGF1-INSR-FOXO3 ligand-receptor-transcription factor axis, a pathway known for enhancing oncogenic resilience and proliferation by inhibiting the transcriptional activity of FOXO3, a key regulator of apoptosis (97–100). Thus, the interplay between glycosyltransferase-enriched macrophages and epithelial cells may drive neoplastic transformation, with the augmented PI3K-Akt pathway potentially supporting the development of M2 macrophages.

Utilizing CellChat, we uncovered a nuanced interaction between macrophage-expressed HLA-B and T-cell-harbored CD8A within the tumor microenvironment, which may be crucial for shaping immunotherapeutic strategies and enhancing immunosurveillance mechanisms. The Major Histocompatibility Complex Class I (MHC-I), including isoforms such as HLA-B, is fundamental in presenting peptide epitopes on the surface of cancer cells, facilitating their recognition by CD8 T cells, and mobilizing their cytotoxic response against malignancies (101–104). Enhanced expression of HLA-B by macrophages could potentially boost the CD8 T-cell-mediated attack on hepatocellular carcinoma (HCC) cells, thereby limiting tumor growth and promoting tumor containment. A notable adaptation in the progression of HCC involves tumor cells evading immune detection by downregulating MHC-I expression (105). However, the increased interaction between HLA-B and CD8A may counteract this evasion, maintaining the immune system’s ability to detect and eliminate cancerous cells. Moreover, the elevated levels of HLA-B and CD8A expression could enhance the antigen presentation process, creating opportunities for refined immunotherapeutic approaches (106). T-cell-based immunotherapies and checkpoint blockade strategies are well-positioned to exploit these molecular interactions, enhancing the effectiveness of immune cells against HCC, promoting tumor control, and stimulating T-cell activation. Additionally, this can catalyze antibody-dependent cellular cytotoxicity (ADCC) within tumors, further contributing to the immune response against HCC.

Utilizing single-cell sequencing stratification, MGAT1 emerged as a pivotal glycosyltransferase-associated hallmark in HCC. Within the glycosyltransferase family, MGAT1 orchestrates secondary N-acetylglucosaminylation, a crucial process in glycoprotein biogenesis (107). A diminished MGAT1 signature in moderately differentiated HCC correlates with tumor dedifferentiation, intrahepatic migration, and a poor clinical prognosis (108).This observation aligns with our spatial-temporal analysis, which showed a decrease in MGAT1 expression from cluster 1 to cluster 6, followed by a resurgence as the transition progressed from cluster 1 to cluster 6 and subsequently to cluster 3. Spatial transcriptomics further supported the hypothesis that macrophages, influenced by HCC and exhibiting low MGAT1 expression, may facilitate HCC dissemination. Murine models lacking MGAT1 revealed increased diacylglycerol (DAG) levels within hepatocytes (109). This lipid second messenger, DAG, activates protein kinase C (PKC), directing it to the cell membrane to regulate key cellular processes such as proliferation and migration (110, 111). MGAT1’s role in forming N-glycosyl moieties also affects the interaction between antibody Fc domains and their receptors, enhancing antibody-dependent cellular cytotoxicity (ADCC) (112–115). Although the current understanding of MGAT1 in HCC is still developing, its detailed investigation is crucial. Our study revealed an increased MGAT1 transcriptional signature in HCC compared to healthy tissues, with this elevation closely linked to a poor HCC prognosis. Our rigorous differential analyses and molecular silencing experiments firmly established MGAT1’s involvement in HCC development, invasion, and metastasis. Therefore, MGAT1’s transcriptional profile in HCC or associated macrophages serves as a promising biomarker, potentially improving diagnostic accuracy and prognostic predictions for HCC patients.

To elucidate MGAT1’s role in HCC immunotherapy, we incorporated an external cohort for an in-depth analysis of Anti-PD-1/PD-L1 checkpoint inhibitors in HCC contexts. These key therapeutic agents, now integral to oncology (116, 117), enhance the immune response against hepatocellular carcinoma by disrupting the PD-1 and PD-L1 interaction (118, 119). This disruption effectively activates T cells, increasing their ability to detect and combat HCC cells (120, 121). Once activated, T cells identify and target HCC cells, leading to their programmed cell death (122, 123). Additionally, Anti-PD-1/PD-L1 agents suppress the unchecked growth and migration of HCC cells, thereby mitigating disease progression (124). Strengthening the immune response, Anti-PD-1/PD-L1 agents effectively inhibit the unrestrained proliferation and migration of HCC cells, thereby altering the course of the disease (125–129). However, evidence suggests that in HCC, PD-L1 phosphorylation at Y112, driven by the IL-6/JAK1 pathway, may create an immune-protected niche. Furthermore, glycosyltransferases in the endoplasmic reticulum play a crucial role in maintaining PD-L1 stability through precise glycosylation. As a result, increased glycosyltransferase activity in HCC might contribute to immune evasion. Interestingly, HCC patients with elevated glycosyltransferase expression may respond better to Anti-PD-1/PD-L1 therapies (74). Thus, the potential of predicting responses to Anti-PD-1/PD-L1 therapy in HCC, influenced by MGAT1 expression, becomes increasingly evident.

In this research, we conducted an extensive examination of glycosyltransferase-associated hallmark genes using both single-cell sequencing and spatial transcriptomics. Single-cell sequencing unveiled subtle expression patterns of glycosyltransferase-linked genes across diverse cellular populations. Concurrently, the innovative spatial transcriptomic technique illuminated the spatial distribution and organization of these genes within the tumor microenvironment. Notably, our study identified a novel HCC-associated glycosyltransferase marker, thereby expanding the toolkit for HCC diagnostics and treatment strategies. This work not only underscores the prognostic significance of glycosyltransferase-signature genes but also advocates for a shift towards personalized therapeutic approaches for high-risk HCC patients.

While our investigation utilizing single-cell and spatial transcriptomic sequencing provides critical insights, several inherent limitations must be acknowledged. Firstly, the amplification and sequencing processes in single-cell protocols may introduce stochastic variations and technical biases, complicating the detection of genes with low expression levels. Differences in sequencing depth and cell capture efficiency further add to interpretative challenges. Secondly, the early development stage of spatial transcriptomic methodologies hinders the decoding of complex tissue architectures. Currently, the spatial resolution of these platforms may not achieve single-cell precision, limiting the ability to obtain cell-specific information in regions with high cellular density. Enhanced data processing and analytical frameworks are required to fully exploit their potential. Furthermore, our analyses were based on publicly available datasets, underscoring the need for prospective studies and extensive *in vivo* and *in vitro* validations. In particular, the glycosyltransferase-associated gene identified in this study warrants thorough investigation in larger, more representative cohorts.

## Data availability statement

The original contributions presented in the study are included in the article/**Supplementary Material**. Further inquiries can be directed to the corresponding author.

## Ethics statement

Ethical approval was not required for the studies on humans in accordance with the local legislation and institutional requirements because only commercially available established cell lines were used.

## Author contributions

YL: Conceptualization, Validation, Visualization, Writing – original draft, Writing – review & editing. YC: Writing – original draft. DW: Writing – original draft. LW: Writing – original draft. TL: Writing – original draft. NA: Writing – original draft. HY: Funding acquisition, Supervision, Writing – original draft, Writing – review & editing.

## Supplementary material

Supplementary Figure 1
**(A)** Immune cell marker gene bubble plot. **(B)** Stromal cell marker gene bubble plot.
