# Supplementary figures and images for: Elucidating the multifaceted role of MGAT1 in hepatocellular carcinoma: integrative single-cell and spatial transcriptomics reveal novel therapeutic insights
Source: Front Immunol. 2024 Jul 16;15:1442722. doi: 10.3389/fimmu.2024.1442722 (PMC11286416; doi:10.3389/fimmu.2024.1442722)

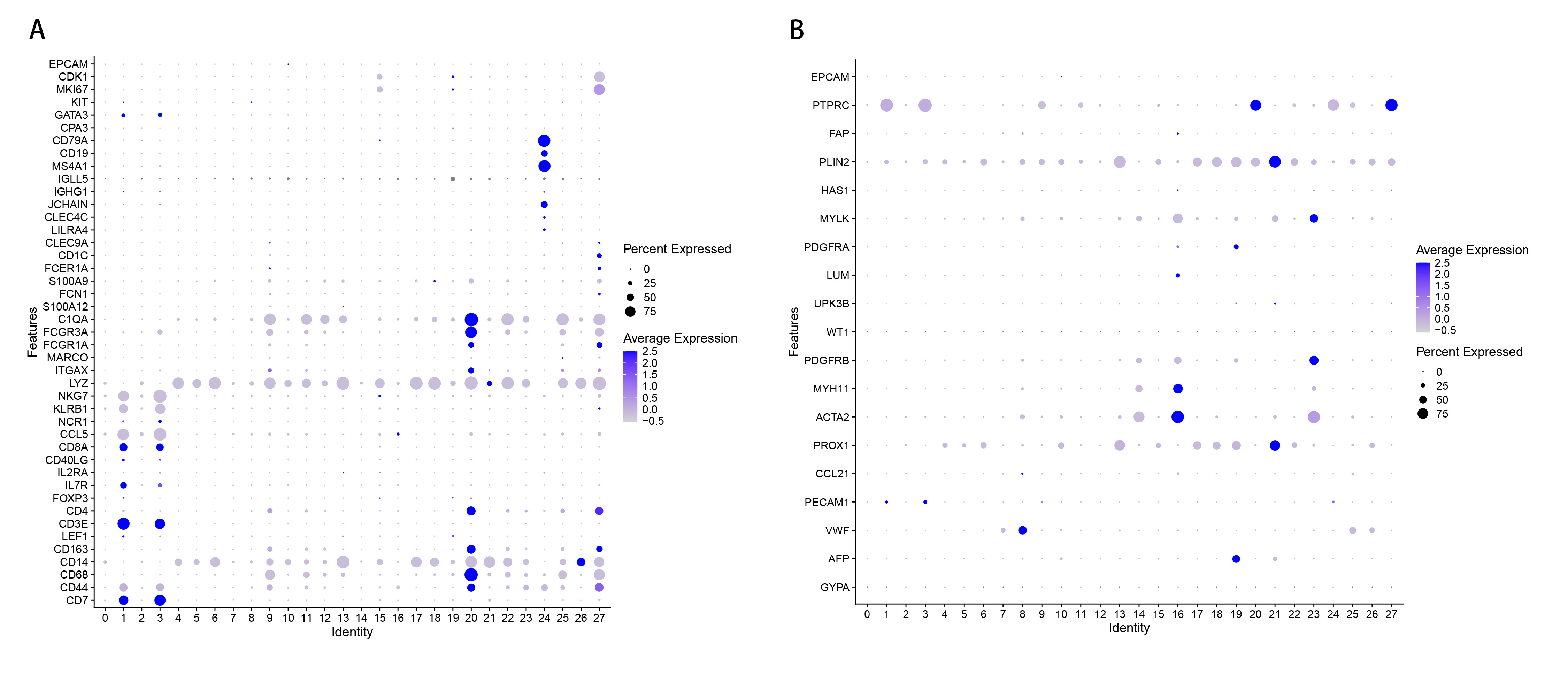

Supplement: Supplementary Figure 1 — (A) Immune cell marker gene bubble plot. (B) Stromal cell marker gene bubble plot. [file Image_1.jpeg]
